# Supplementary material for: High-throughput screening of BAM inhibitors in native membrane environment
Source: Nat Commun. 2023 Sep 13;14:5648. doi: 10.1038/s41467-023-41445-w (PMC10499997; doi:10.1038/s41467-023-41445-w)
Supplement: Supplementary file 1 — Supplementary Information [file 41467_2023_41445_MOESM1_ESM.pdf]

## Supplementary Figures

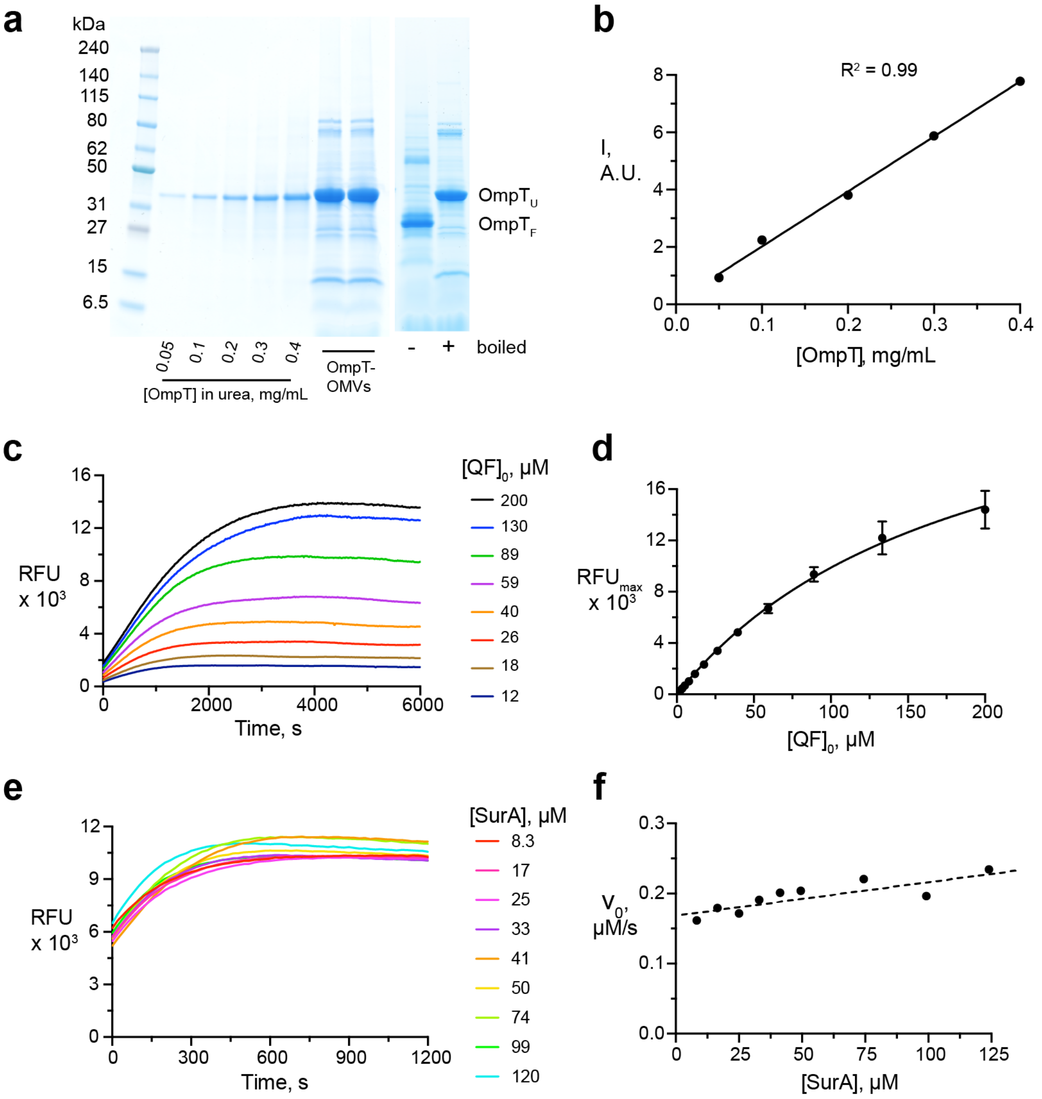


**Supplementary Figure 1. Characterization of OmpT-OMVs.** (**a**) SDS-PAGE of unboiled and boiled OmpT-OMVs along with pure OmpT in urea solution at known concentrations. The uncropped gel is shown in Supplementary Figure 10. (**b**) Linear fit to calibrate band intensities. (**c**) Fluorescence emitted after the cleavage of QF peptide by OmpT-OMVs using variable $[QF]_0$. (**d**) Scaling curve obtained from fitting the maximum fluorescence values from **c**. n=3 independent experiments. Data are presented as average values ± standard deviation. (**e**) Progress curves of the cleavage of 100 μM QF peptide by 8.4 nM OmpT-OMV at variable SurA concentrations as indicated. (**f**) Initial reaction velocities from **e**, plotted as a function of chaperone concentration. The dashed line is a linear regression to the data. The data underlying panels **b–f** are provided as Source Data.

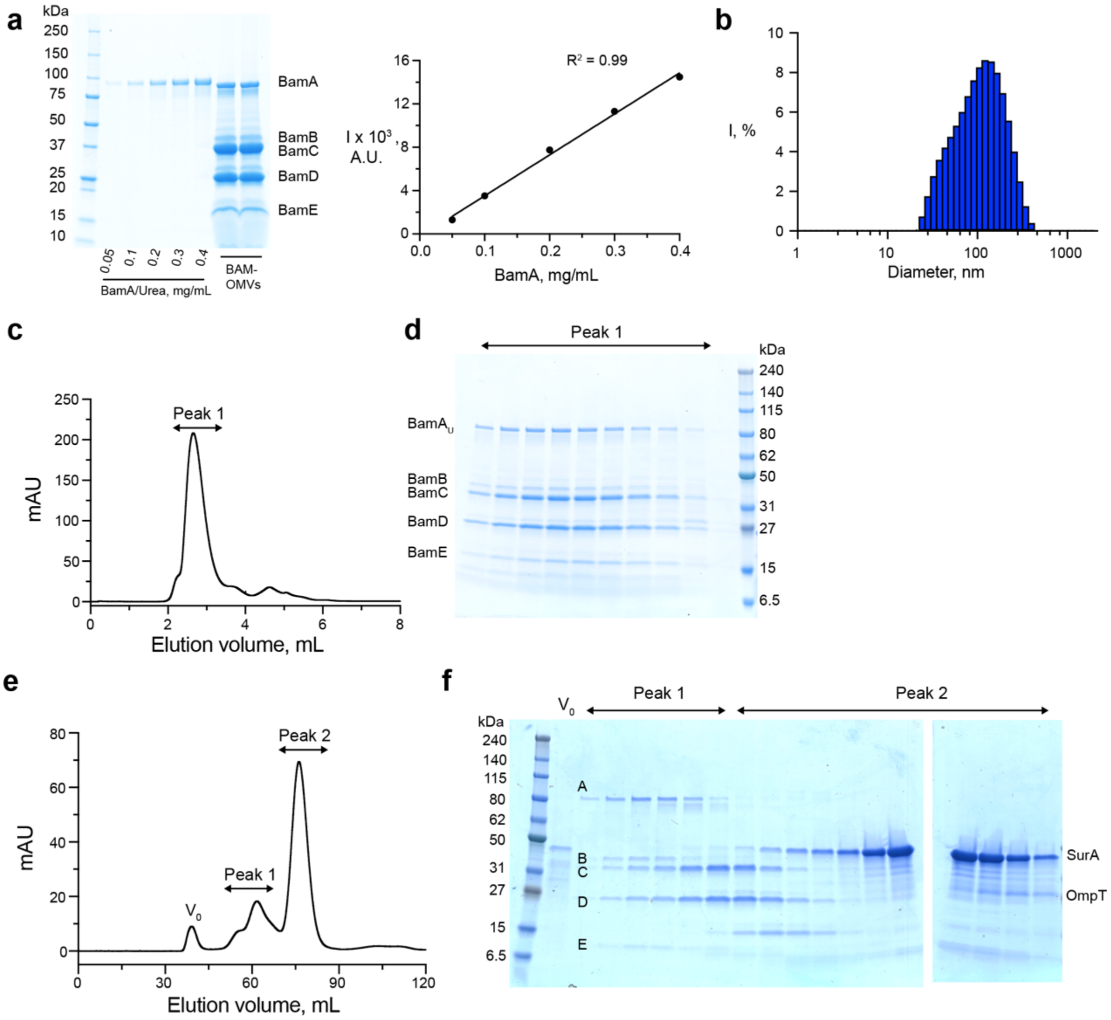


**Supplementary Figure 2. Characterization of BAM-OMVs.** (**a**) SDS-PAGE of BAM-OMVs along with pure BamA in urea at known concentrations and a linear fit to calibrate band intensities. The uncropped gel is shown in Supplementary Figure 11. (**b**) Size distribution of OMVs as observed by dynamic light scattering (DLS). (**c**) Size exclusion chromatography characterization of BAM complex extracted from BAM-OMVs with 1% DDM on a manually packed 4 mL superdex S200 column equilibrated in 20 mM Tris-Cl, pH 8.0, 150 mM NaCl containing 0.05% DDM. (**d**) SDS-PAGE of elution fractions from peak 1 demonstrating individual component of BAM complex. Note that a majority of the peak volume comprises complete BAM complexes and only a minority corresponds to excess BamC and BamD. The uncropped gel is shown in Supplementary Figure 12. (**e**) SEC elution profile of a DDM-extracted reaction mixture comprising BAM-OMVs, SurA, urea-unfolded OmpT and colistin. Two separate elution peaks were observed besides the void volume peak ($V_0$). (**f**) SDS-PAGE of elution fractions from peak 1 and 2, demonstrating intact BAM complex and SurA-OmpT. The uncropped gels are shown in Supplementary Figure 13. The data underlying panels **a**–**c** and **e** are provided as Source Data.

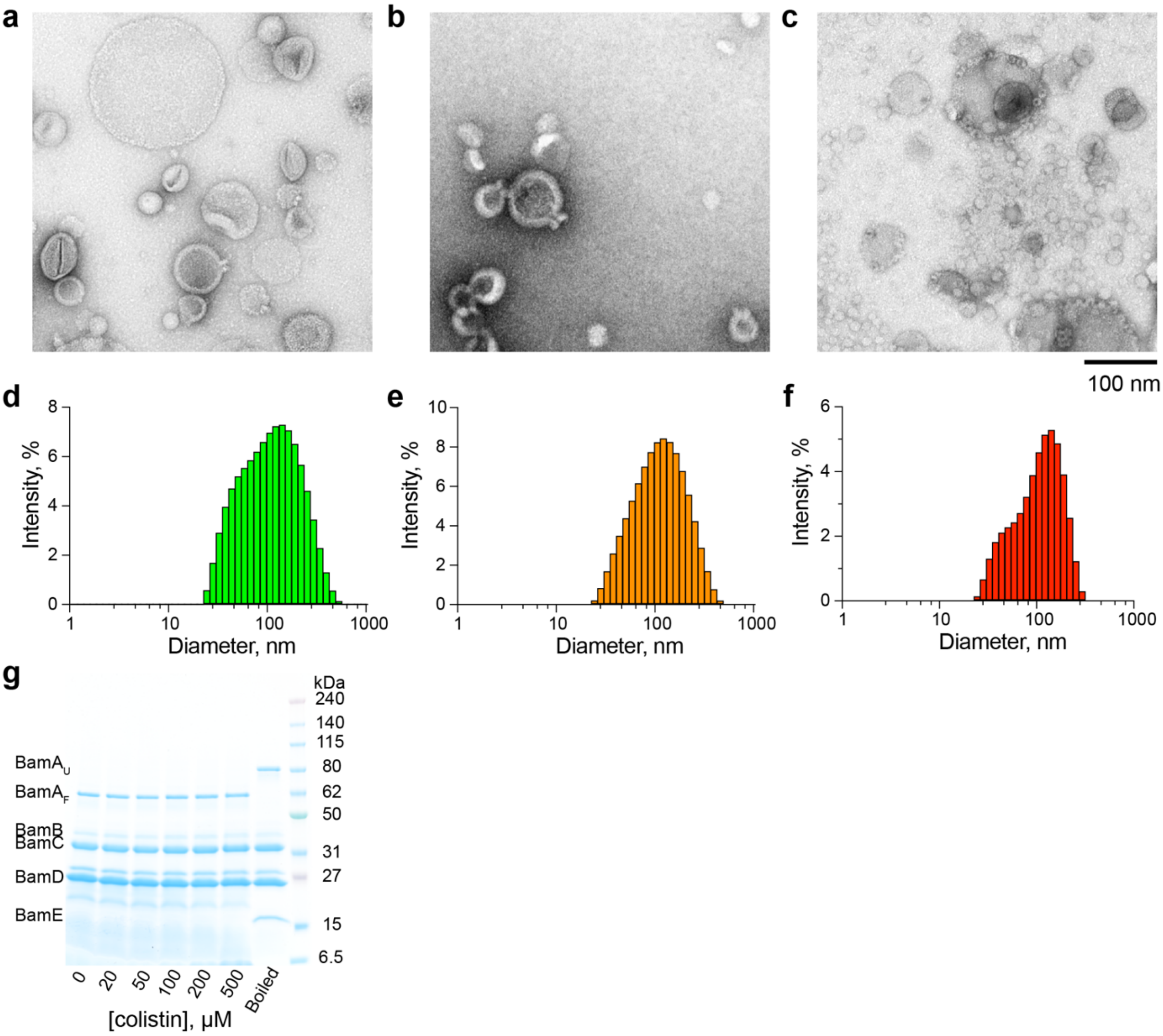


**Supplementary Figure 3.** Negative staining electron micrographs of BAM-OMVs incubated with variable colistin concentrations. (**a**) 20 μM, (**b**) 50 μM, and (**c**) 100 μM colistin. (**d**–**f**) The average size distribution of OMVs at (**d**) 20 μM, (**e**) 50 μM, and (**f**) 100 μM colistin as observed by DLS measurement. (**g**) SDS-PAGE gel-shift assay of BAM-OMVs treated with colistin concentrations up to 500 μM, demonstrating proper folding of BamA at all concentrations. The uncropped gel is shown in Supplementary Figure 14. The data underlying panels **d**–**f** are provided as Source Data.

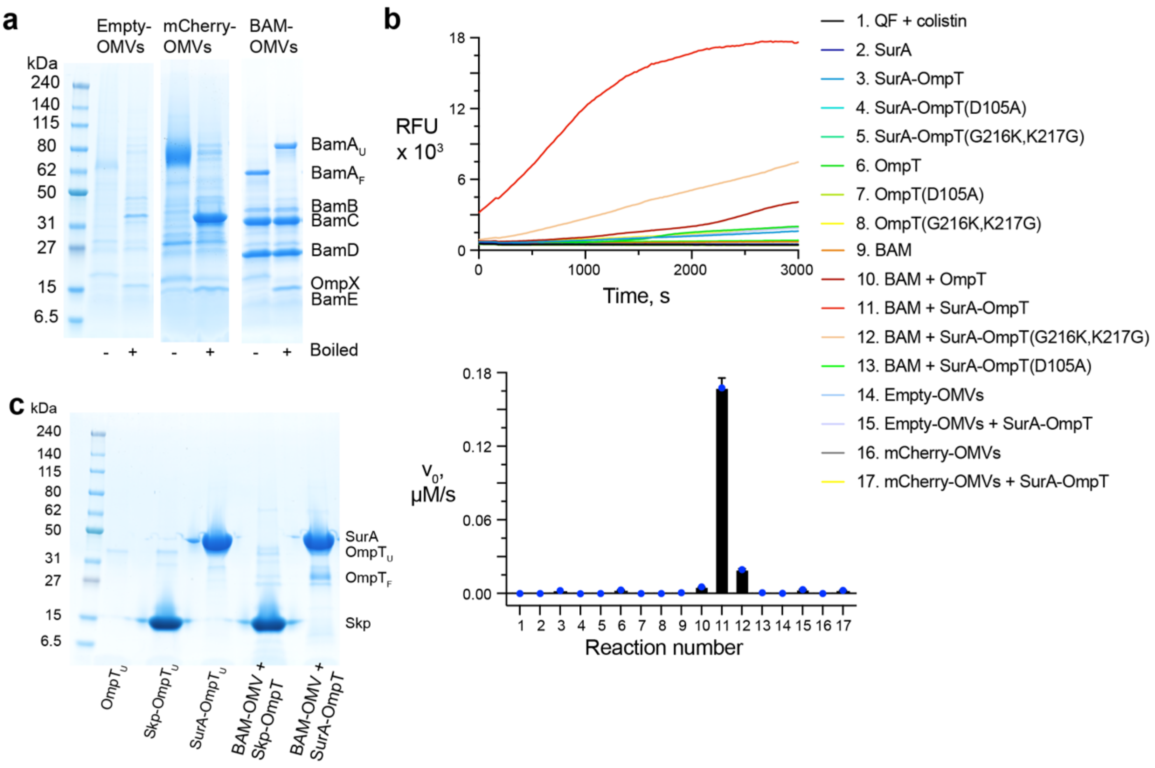


**Supplementary Figure 4. BAM-mediated OmpT folding with control reactions.** (**a**) SDS-PAGE analysis of empty-OMVs, mCherry-OMVs, and BAM-OMVs. The BAM complex is not detectable in empty OMVs and mCherry OMVs, but is present in BAM-OMVs. The strong band in mCherry-OMVs at 70 kDa/35 kDa for -/+ boiling is the mCherry dimer and monomer, respectively. The uncropped gel is shown in Supplementary Figure 15. (**b**) Fluorescence emission progress curves of cleavage of QF peptide in presence of variable reaction components 1–17, as indicated, and bar graph of the resulting initial reaction velocities $v_0$. n=2 independent experiments. Data are presented as average values ± standard deviation. (**c**) SDS-PAGE of unboiled reaction mixtures demonstrating the presence of folded OmpT ($OmpT_F$) in BAM-OMVs, starting from unfolded OmpT ($OmpT_U$) bound to the chaperones Skp or SurA. The uncropped gel is shown in Supplementary Figure 16. The data underlying panel **b** are provided as Source Data.

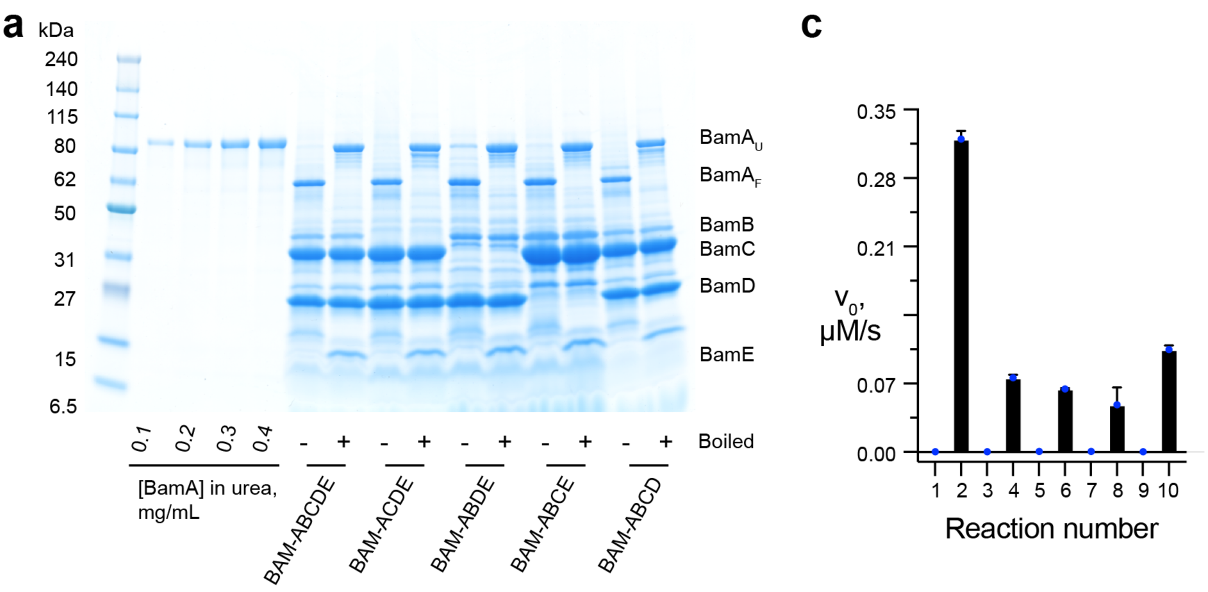


**Supplementary Figure 5. Effect of BAM lipoproteins on BAM-mediated OmpT folding in OMVs.** (**a**) SDS-PAGE of different preparations of BAM lipoprotein knockouts in OMVs along with pure BamA in urea at known concentrations. The uncropped gel is shown in Supplementary Figure 17. (**b**) Fluorescence emission of BAM-mediated OmpT folding of different reaction mixtures 1–10 as mentioned. (**c**) Initial reaction velocities for different reaction mixes 1–10 as indicated in **b**. n=2 independent experiments. Data are presented as average values ± standard deviation. The data underlying panels **b**–**c** are provided as Source Data.

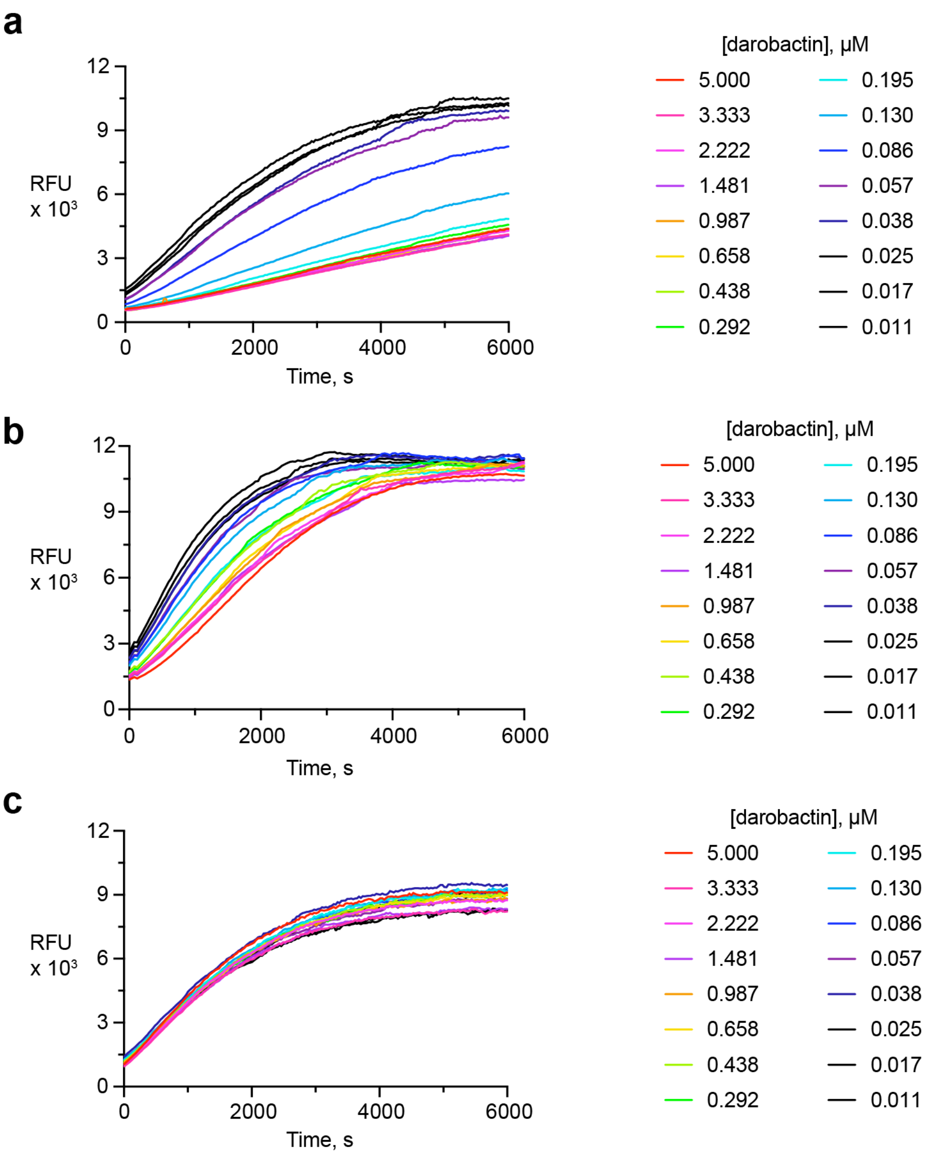


**Supplementary Figure 6. Effect of darobactin on BAM-mediated folding of OmpT in OMVs.** Fluorescence emission of BAM-mediated OmpT folding at variable darobactin concentration, as indicated, in (**a**) presence of colistin and (**b**) absence of colistin with sonicated BAM-OMVs. (**c**) Peptidase activity of OmpT in OmpT-OMVs at variable darobactin concentration, as indicated. The data underlying panels **a**–**c** are provided as Source Data.

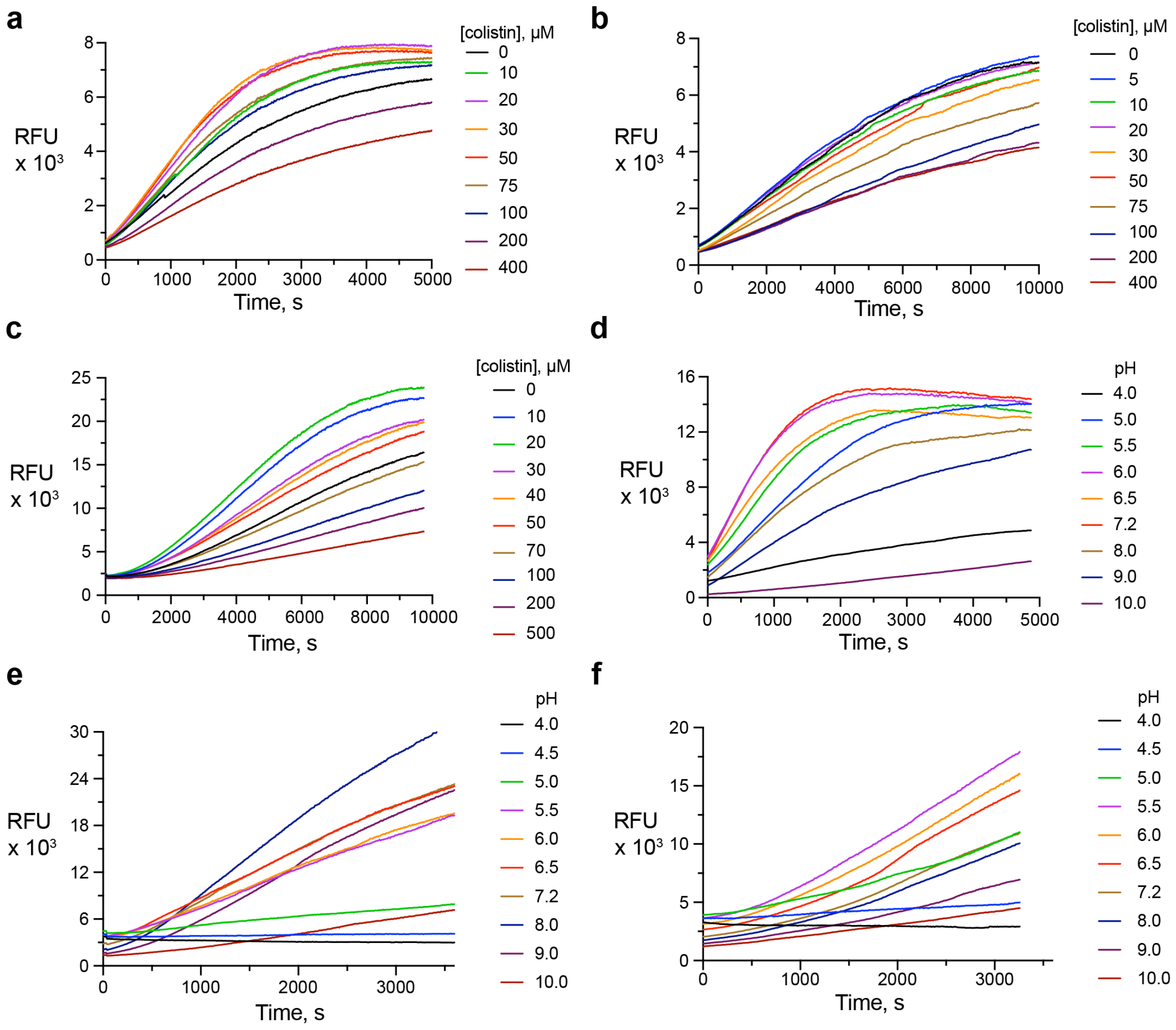


**Supplementary Figure 7. Fluorescence emission for optimization of colistin concentration and reaction buffers.** (**a**–**c**) Fluorescence emission progress curves of BAM-mediated OmpT activity in presence of (**a**) reaction mixes comprised of 16 nM BAM, 100 μM QF, 100 μm SurA and 2 μm OmpT and variable colistin concentration, as indicated, (**b**) reaction mixes comprised of sonicated BAM OMVs with 16 nM BAM, 100 μM QF, 100 μm SurA and 2 μm OmpT and variable colistin concentration, as indicated. (**c**) Reaction mixes comprised of 16 nM BAM, 500 μM QF, 50 μm Skp and 2 μm OmpT and variable colistin concentration, as indicated. (**d**) Reaction mixes comprised of 0.8 nM OmpT, 100 μM QF, 50 μm colistin at variable pH, as indicated. (**e**) Reaction mixes comprised of 16 nM BAM, 500 μM QF, 50 μm colistin, 50 μm SurA and 2 μm OmpT at variable pH, as indicated. (**f**) Reaction mixes comprised of 16 nM BAM, 500 μM QF, 50 μm colistin, 50 μm Skp and 2 μm OmpT at variable pH, as indicated. The initial reaction velocities from these experiments are shown in Figure 3. The data underlying panels **a**–**f** are provided as Source Data.

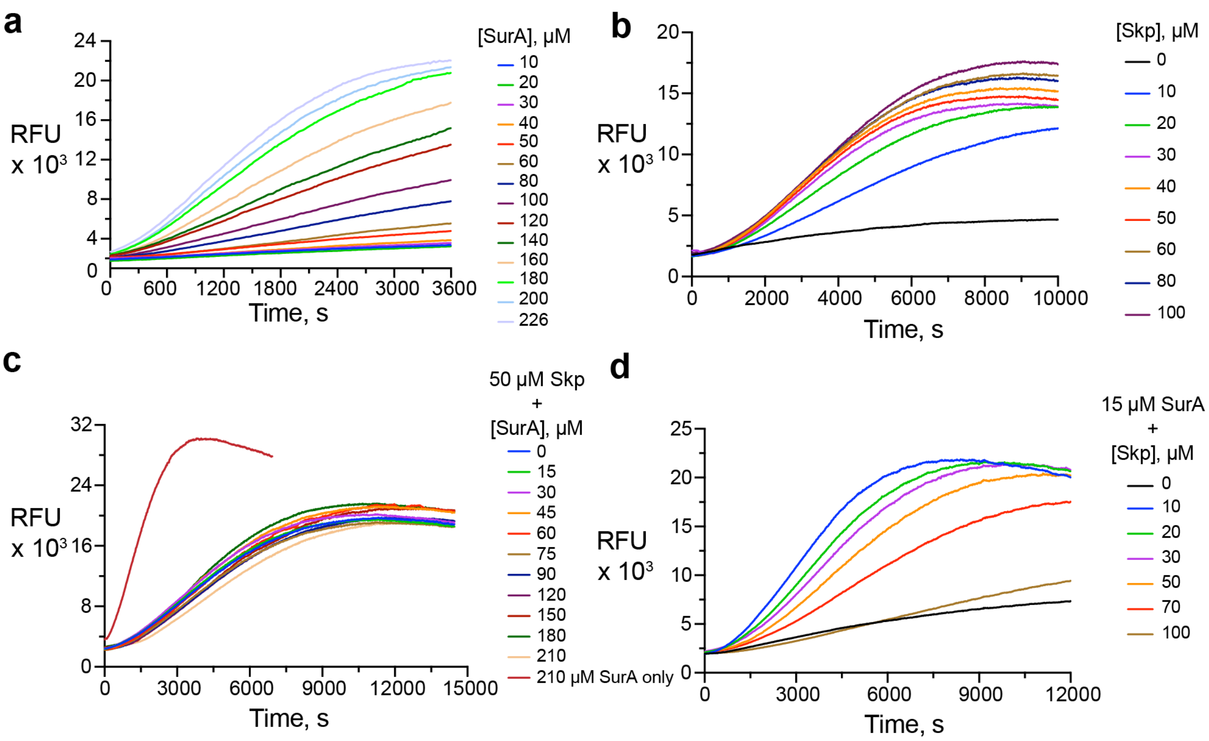


**Supplementary Figure 8. Optimization of chaperone concentration and their competitive effect on BAM-mediated OmpT folding.** (**a**, **b**) Fluorescence emission of BAM-mediated OmpT folding at variable (**a**) SurA and (**b**) Skp concentrations, as indicated. (**c**) Fluorescence emission of BAM-mediated OmpT activity at 50 μM Skp and variable SurA concentration, as indicated. (**d**) Fluorescence emission of BAM-mediated OmpT activity at 15 μM SurA and variable Skp concentration, as indicated. Each reaction mix comprised of 16 nM BAM in OMVs, 20 μM colistin, 500 μM QF peptide, 2 μM unfolded OmpT in addition to Skp and SurA at the indicated concentrations. The initial reaction velocities from these experiments are shown in Figure 3. The data underlying panels **a**–**d** are provided as Source Data.

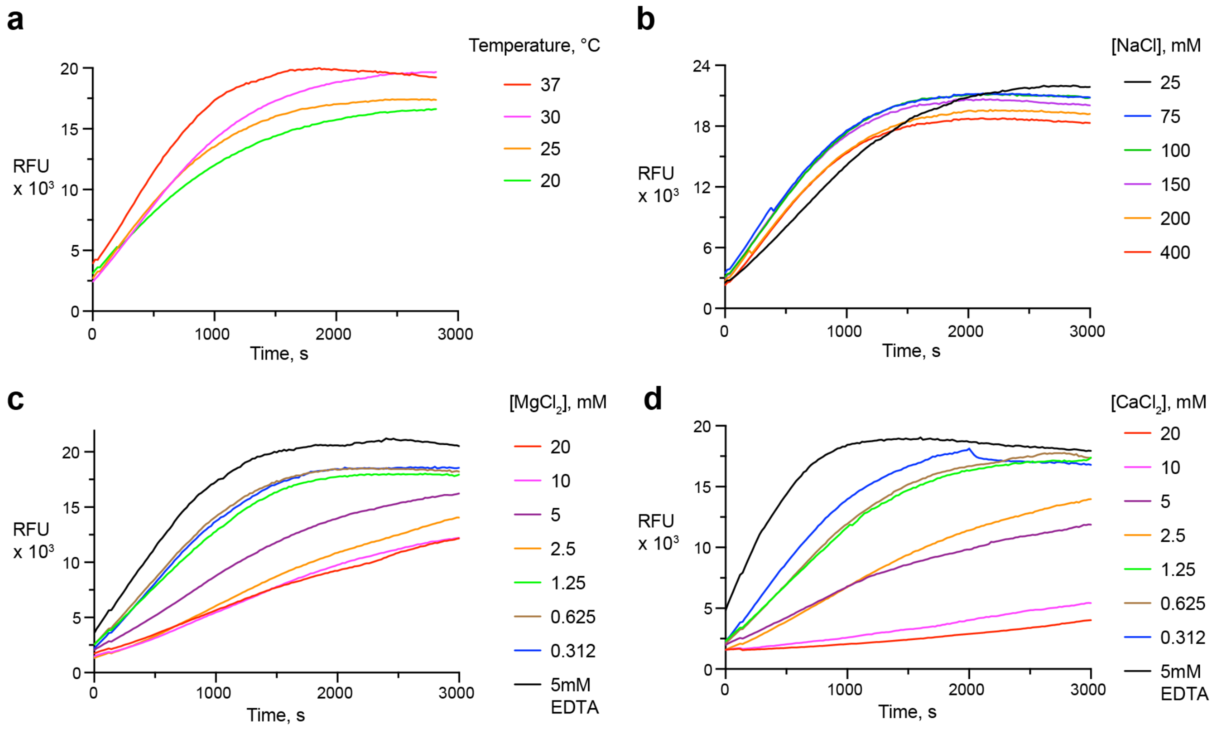


**Supplementary Figure 9. Fluorescence emission for optimization of reaction parameters for BAM-mediated OmpT folding.** Fluorescence emission of BAM-mediated OmpT folding at variable (**a**) reaction temperatures, (**b**) NaCl, (**c**) $MgCl_2$, and (**d**) $CaCl_2$ concentrations as indicated. The reaction mixture comprised of 100 nM BAM in OMVs, 20 µM colistin, 100 µM QF peptide, 100 µm SurA and 2 µm OmpT. The initial reaction velocities from these experiments are shown in Figure 3. The data underlying panels **a**–**d** are provided as Source Data.

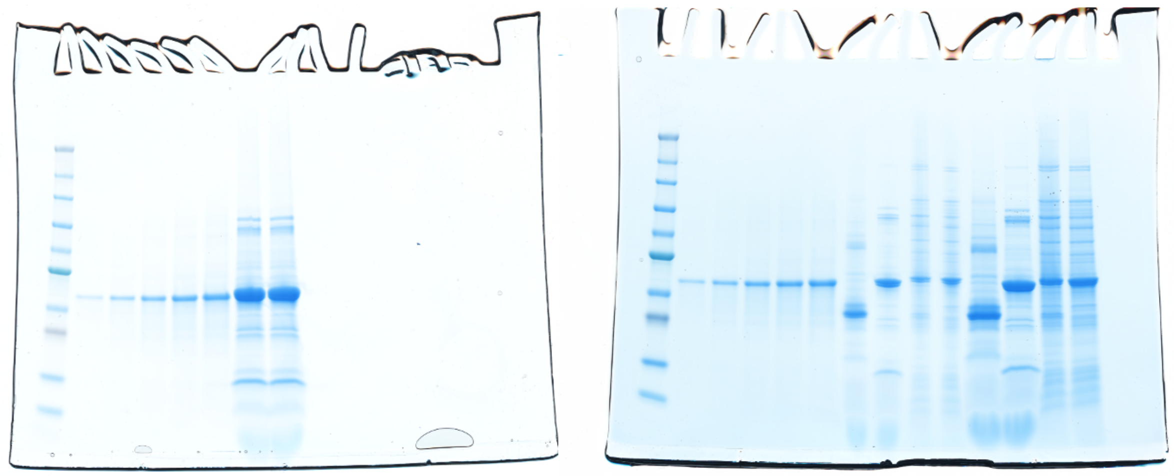

**Supplementary Figure 10. Uncropped gels of Supplementary Figure 1a.** Coomassie Blue stained 4–20% SDS-PAGE with molecular weight standard in the first lane.

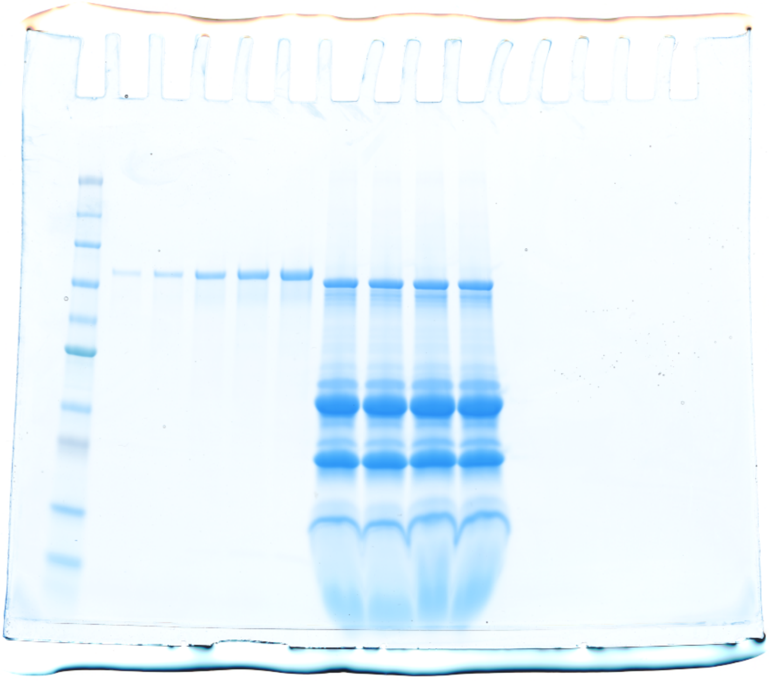

**Supplementary Figure 11. Uncropped gel of Supplementary Figure 2a.** Coomassie Blue stained 4–20% SDS-PAGE with molecular weight standard in the first lane.

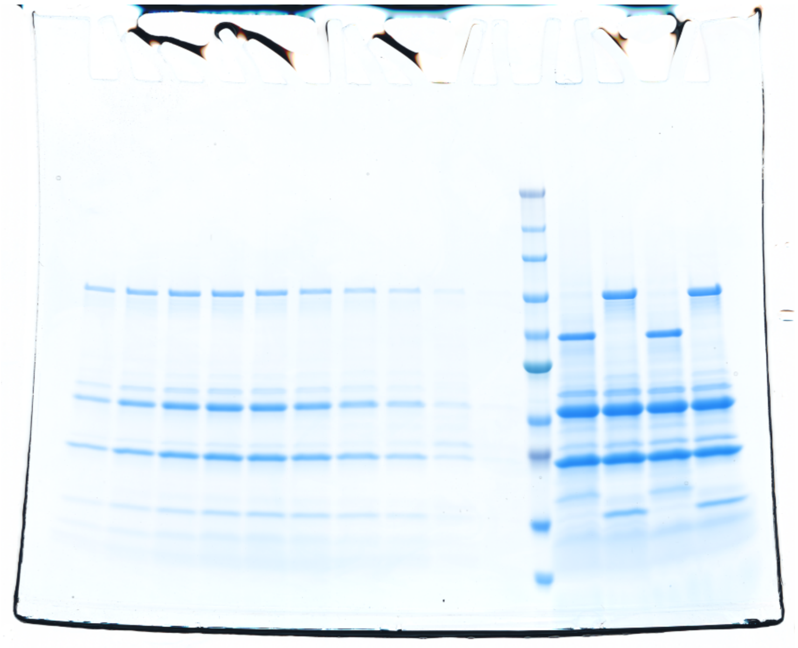

**Supplementary Figure 12. Uncropped gel of Supplementary Figure 2d.** Coomassie Blue stained 4–20% SDS-PAGE with molecular weight standard in lane 11.

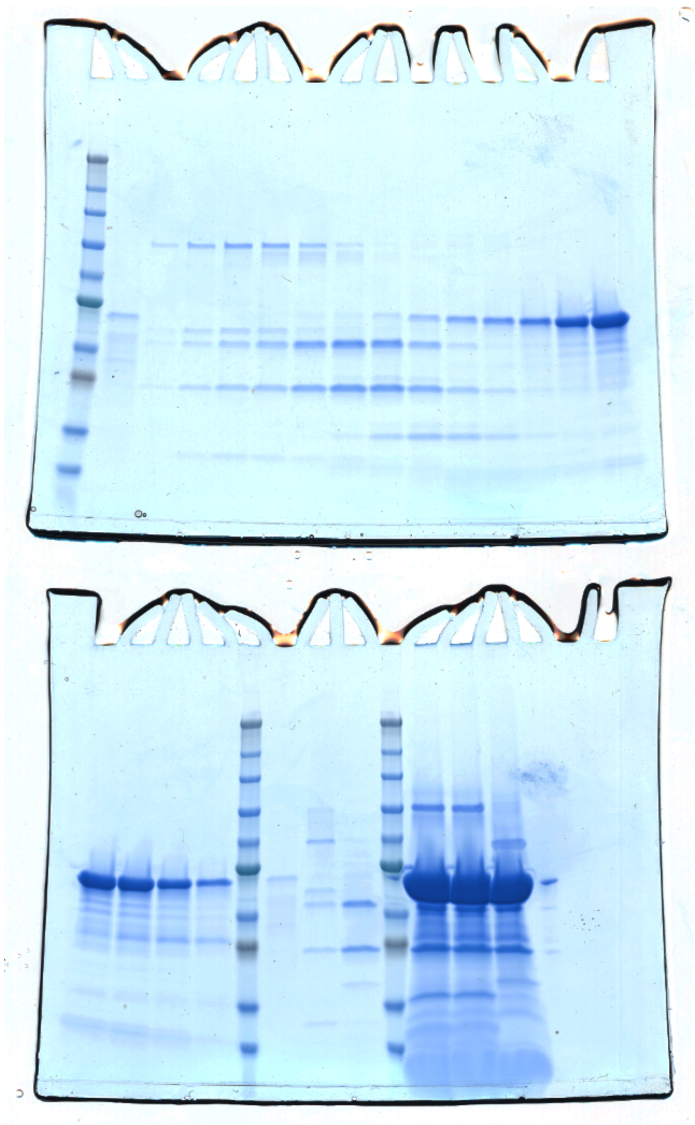

**Supplementary Figure 13. Uncropped gels of Supplementary Figure 2f.** Coomassie Blue stained 4–20% SDS-PAGE with molecular weight standard in lanes 1 / lanes 5 & 9.

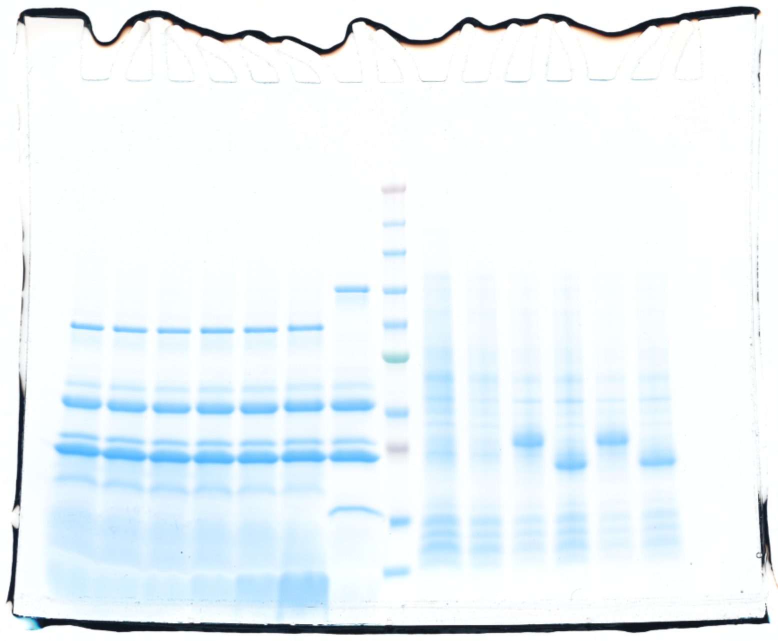

**Supplementary Figure 14. Uncropped gel of Supplementary Figure 3g.** Coomassie Blue stained 4–20% SDS-PAGE with molecular weight standard in lane 8.

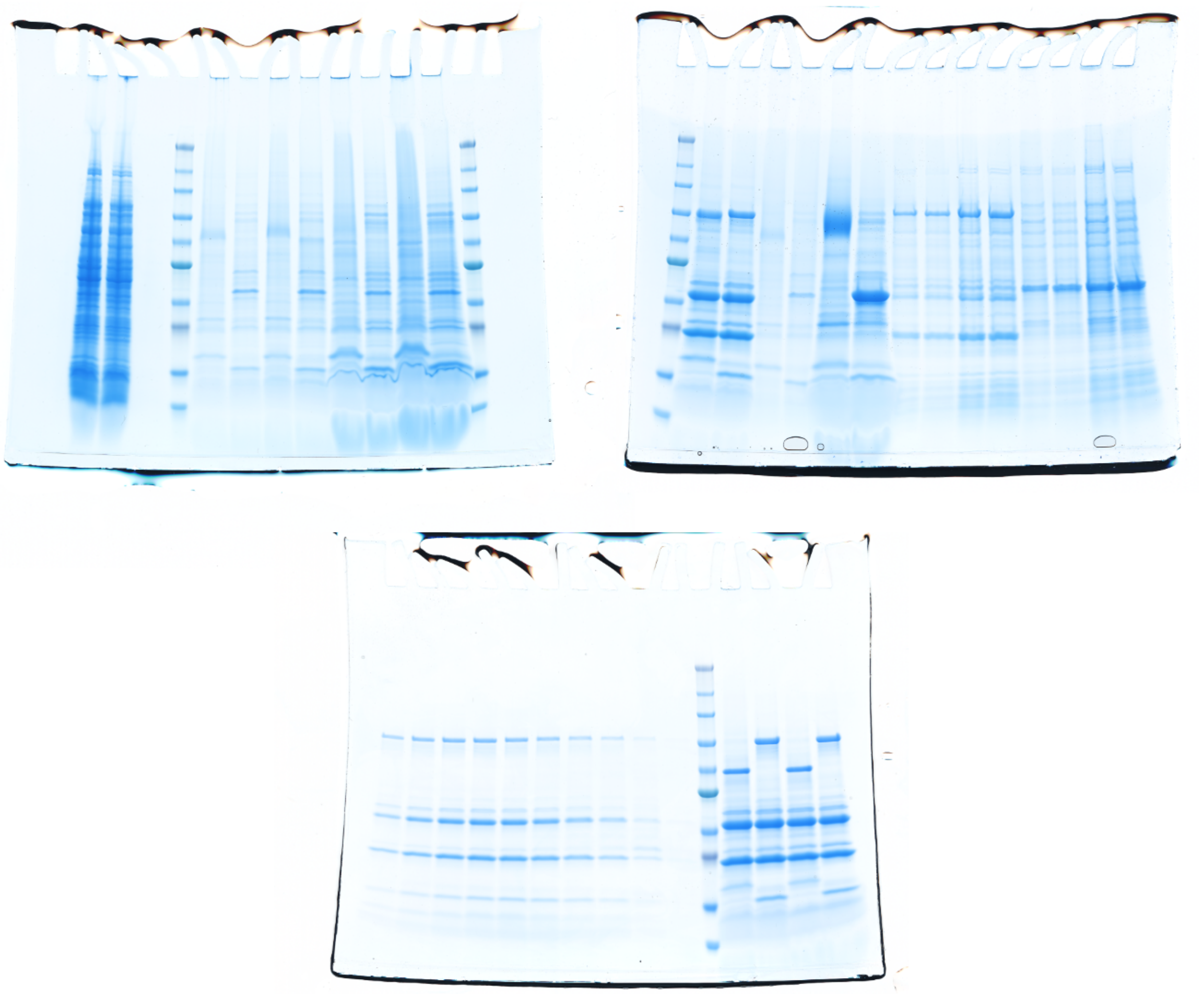

**Supplementary Figure 15. Uncropped gels of Supplementary Figure 4a.** Coomassie Blue stained 4–20% SDS-PAGEs with molecular weight standards in lanes 5 & 14 / lane 1 / lane 11. Top left: Empty-OMVs, top right: mCherry-OMVs, bottom: BAM-OMVs.

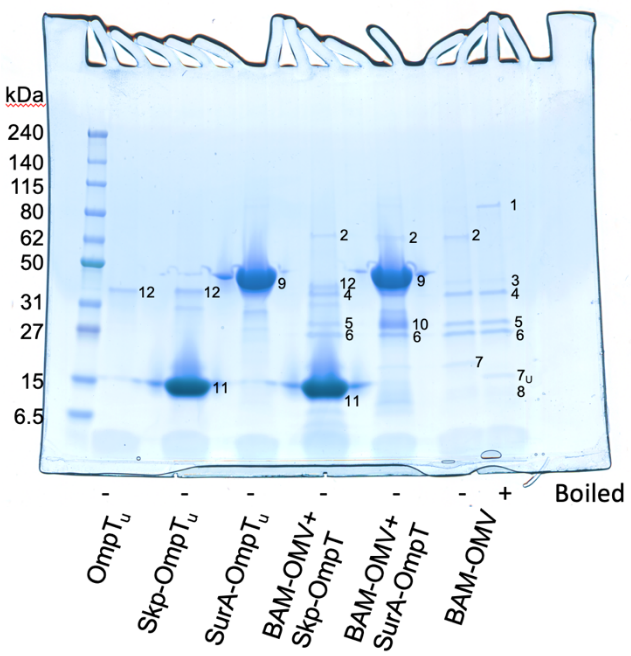


1. Unfolded BamA (88 kDa)
2. Folded BamA (apparent mass 60–70 kDa)
3. BamB (44 kDa; faint)
4. BamC (36 kDa)
5. Truncated BamC (identified by Mass Spectrometry)
6. BamD (27 kDa)
7. OmpX (identified by Mass Spectrometry)
8. BamE (13 kDa; faint)
9. SurA (45 kDa)
10. Folded OmpT (apparent mass 25–30 kDa)
11. Skp (17 kDa)
12. Unfolded OmpT (35 kDa)

**Supplementary Figure 16. Uncropped gel of Supplementary Figure 4c.** Coomassie Blue stained 4–20% SDS-PAGE with molecular weight standard in the first lane. Samples were loaded with or without boiling, as indicated below the gel. Annotations of all bands are given on the right.

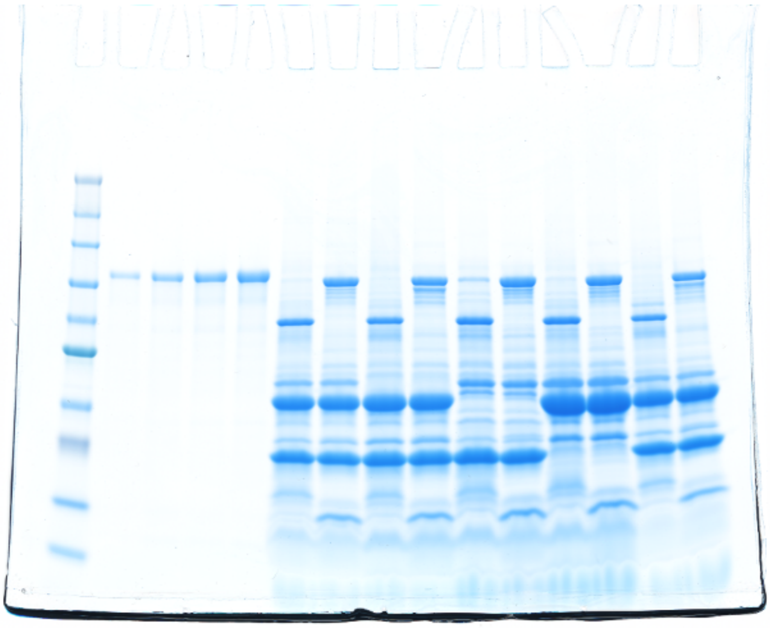

**Supplementary Figure 17. Uncropped gel of Supplementary Figure 5a.** Coomassie Blue stained 4–20% SDS-PAGE with molecular weight standard in the first lane.
