# Supplementary material for: High-throughput screening of BAM inhibitors in native membrane environment
Source: Nat Commun. 2023 Sep 13;14:5648. doi: 10.1038/s41467-023-41445-w (PMC10499997; doi:10.1038/s41467-023-41445-w)
Supplement: Supplementary file 3 — Reporting Summary [file 41467_2023_41445_MOESM3_ESM.pdf]

nature portfolio

Corresponding author(s): Sebastian Hiller, Christoph Bieniossek

Last updated by author(s): Aug 31, 2023

Reporting Summary

Nature Portfolio wishes to improve the reproducibility of the work that we publish. This form provides structure for consistency and transparency in reporting. For further information on Nature Portfolio policies, see our Editorial Policies and the Editorial Policy Checklist.

Statistics

For all statistical analyses, confirm that the following items are present in the figure legend, table legend, main text, or Methods section.

n/a	Confirmed	
☐	☒	The exact sample size (*n*) for each experimental group/condition, given as a discrete number and unit of measurement
☐	☒	A statement on whether measurements were taken from distinct samples or whether the same sample was measured repeatedly
☒	☐	The statistical test(s) used AND whether they are one- or two-sided<br>*Only common tests should be described solely by name; describe more complex techniques in the Methods section.*
☒	☐	A description of all covariates tested
☒	☐	A description of any assumptions or corrections, such as tests of normality and adjustment for multiple comparisons
☐	☒	A full description of the statistical parameters including central tendency (e.g. means) or other basic estimates (e.g. regression coefficient) AND variation (e.g. standard deviation) or associated estimates of uncertainty (e.g. confidence intervals)
☒	☐	For null hypothesis testing, the test statistic (e.g. *F*, *t*, *r*) with confidence intervals, effect sizes, degrees of freedom and *P* value noted<br>*Give P values as exact values whenever suitable.*
☒	☐	For Bayesian analysis, information on the choice of priors and Markov chain Monte Carlo settings
☒	☐	For hierarchical and complex designs, identification of the appropriate level for tests and full reporting of outcomes
☒	☐	Estimates of effect sizes (e.g. Cohen's *d*, Pearson's *r*), indicating how they were calculated

Our web collection on statistics for biologists contains articles on many of the points above.

Software and code

Policy information about availability of computer code

Data collection	Titan Krios 300 keV electron microscope (EFI), equipped with GF Quantum LS Imagining filter (Gatan) and K2 summit (Gatan), operated with software EM version number 3.8; Tecan Spark multi-mode microplate reader operateed with software SparkControl, version number V2.3.
Data analysis	Microsoft Excel version MS16.75; Graphpad Prism version 8.4.3 (686).

For manuscripts utilizing custom algorithms or software that are central to the research but not yet described in published literature, software must be made available to editors and reviewers. We strongly encourage code deposition in a community repository (e.g. GitHub). See the Nature Portfolio guidelines for submitting code & software for further information.

Data

Policy information about availability of data

All manuscripts must include a data availability statement. This statement should provide the following information, where applicable:

- Accession codes, unique identifiers, or web links for publicly available datasets
- A description of any restrictions on data availability
- For clinical datasets or third party data, please ensure that the statement adheres to our policy

The experimental data that support the findings of this study is shown in the article and its supplementary materials. The raw data underlying all Figures and Supplementary Figures is provided as a Source Data file.

Human research participants

Policy information about studies involving human research participants and Sex and Gender in Research.

Reporting on sex and gender	NA
Population characteristics	NA
Recruitment	NA
Ethics oversight	NA

Note that full information on the approval of the study protocol must also be provided in the manuscript.

Field-specific reporting

Please select the one below that is the best fit for your research. If you are not sure, read the appropriate sections before making your selection.

- [x] Life sciences
- [ ] Behavioural & social sciences
- [ ] Ecological, evolutionary & environmental sciences

For a reference copy of the document with all sections, see nature.com/documents/nr-reporting-summary-flat.pdf

Life sciences study design

All studies must disclose on these points even when the disclosure is negative.

Sample size	No statistical method was used to determine sample sizes and no sample size calculation was performed. The standard in the research field is to sample n>=3 independent measurements. Consequently, at least three independent replicates were performed for each condition and/or titration series with more than three data points.
Data exclusions	No data was excluded
Replication	Reported results were consistently replicated across multiple experiments. All replicates generated similar results. All experiments were carried out at least in triplicate and/or titration series with more than three data points, and the number of independent replicates is specified in each Figure caption.
Randomization	Randomization was not necessary, because all experimental parameters were precisely controlled and the experiments were performed with standardized ingredients. Randomization is typically not used in this field.
Blinding	Investigators were not blinded. Blinding during data collection was not needed because all experimental conditions were precisely controlled. Blinding during the analysis was not needed because the data was recorded by instruments, the results were quantitative and not subject to individual judgement or interpretation. Blinding is typically not used in this field.

Reporting for specific materials, systems and methods

We require information from authors about some types of materials, experimental systems and methods used in many studies. Here, indicate whether each material, system or method listed is relevant to your study. If you are not sure if a list item applies to your research, read the appropriate section before selecting a response.

Materials & experimental systems

n/a	Involved in the study	
x		Antibodies
x		Eukaryotic cell lines
x		Palaeontology and archaeology
x		Animals and other organisms
x		Clinical data
x		Dual use research of concern

Methods

n/a	Involved in the study	
x		ChIP-seq
x		Flow cytometry
x		MRI-based neuroimaging
